# How to Ask the Right Question and Find the Right Answer: Clinical Research for Transplant Nephrologists

**DOI:** 10.3389/fimmu.2022.879200

**Published:** 2022-05-10

**Authors:** Sonia Rodríguez-Ramírez, S. Joseph Kim

**Affiliations:** ^1^Division of Nephrology, University Health Network, Toronto, ON, Canada; ^2^Ajmera Transplant Centre, University Health Network, Toronto, ON, Canada; ^3^Institute of Health Policy, Management and Evaluation, University of Toronto, Toronto, ON, Canada

**Keywords:** clinical research, randomized control trial, cohort study, case-control study, cross-sectional study, case report

## Abstract

Clinical research is about asking and answering questions. Before solutions relevant to clinical problems can be sought, clinicians must frame questions in ways that are answerable using the methods of clinical research. Different types of questions are best answered using specific study designs. Each design has inherent strengths and limitations. In this review article, we provide an approach to asking answerable clinical research questions, review the major study designs, describe their strengths and weaknesses, and link the study designs to their intended purposes.

## Introduction

Clinical transplantation has developed in parallel with many companion sciences and has been the initial testing ground for numerous novel surgical techniques, medications, and clinical practice. This close association with medical advancement has required an almost continuous relationship between clinical transplantation and clinical research. In order to generate appropriate data summaries and inferences about any population, it is necessary to utilize the appropriate methods (1–4). Evidence-based medicine aims to inform such questions with the judicious use of the best available research (5).

Before solutions relevant to clinical problems can be sought, one must frame questions in ways that are answerable, using the methods of clinical research. A well formulated question is the half the battle. Thereafter, an approach to answering the question has to be formulated. Different types of questions are best answered using specific study designs. Each design has its inherent strengths and limitations for addressing the objectives of the study. However, recognizing different study designs and choosing the most appropriate one for a given question is not always straightforward.

The purpose of this article is to outline an approach to asking answerable clinical research questions, to review the major study designs, to describe the strengths and limitations of each design, and to link the study designs to their intended purposes. Reviewing specific statistical approaches to analyzing clinical research studies is beyond the scope of this article but we have provided several excellent resources to which the reader can refer (6–8).

## Asking The Right Question

The first step in discovering new insights on an issue relevant to patient care is framing the question so that it is answerable using the methods of clinical research (5). The PICO model is an evidence-based model for formulating a clinical research question (9, 10):

- Patient, population, or problem (P): Which characteristics, like the target clinical condition, ethnicity, and age group, define the patients or population?
- Intervention (I): Which intervention or exposure (e.g., form of treatment, diagnostic test, or educational program) is being applied to the patient/population/problem?
- Comparator or control (C): Is there an alternative to the main intervention, for example, treatment with placebo or the standard of care? This category is only applicable to studies with a comparator group (i.e., analytical studies).
- Outcomes or effects (O): Which outcomes or effects relating to the intervention or exposure (e.g., mortality, morbidity, quality of life, cost-effectiveness) are being studied?

Framing the clinical question determines the question type (etiology, diagnosis, prognosis, intervention), which then determines the most appropriate study design to answer the question (**Table 1**). For example, in adult patients with end-stage kidney disease from primary focal segmental glomerulosclerosis who have received a kidney transplant (P), does bilateral native nephrectomy (I), when compared with conservative treatment (C), reduce the risk of disease recurrence after kidney transplant (O)?

**Table 1:** Appropriate study design for addressing different clinical questions.

Question type	Example	Best study design	Other possible designs
**Intervention**	Drug vs. control Drug vs. drug Procedure vs. control	Randomized controlled trial	Cohort study Case-control study
**Diagnostic accuracy**	Test A vs. reference test Test A vs. test B vs. reference	Cross-sectional study	–
**Etiology**	Exposure present vs. absent	Cohort study	Case-control study
**Prognosis**	Exposure present vs. absent	Cohort study	–
**Descriptive**	Period prevalence*	Cohort study	–
	Point prevalence**	Cross-sectional	–
	Incidence	Cohort	–

Adapted from Cross NB, Craig JC, Webster AC. Asking the right question and finding the right answers. Nephrology (Carlton). 2010 Feb;15(1):8-11. doi: 10.1111/j.1440-1797.2009.01264.x. PMID: 20377764.

*Period prevalence refers to prevalence measured over an interval of time.

**Point prevalence refers to the prevalence measured at a particular point in time. It is the proportion of persons with a particular disease or attribute on a particular date.

Along with clarifying the objectives of a research project, the PICO method directly supports electronic search strategies on platforms such as PubMed. An extension of PICO adds a “T” for time frame and “S” for setting (i.e., PICOTS). The latter version may be particularly useful for observational studies that use existing data sources. Alternative approaches to framing research questions exist and may be appropriate for specific circumstances (e.g., ECLIPSE for qualitative research) (11, 12).

## Finding The Right Answer

**Figure 1:**
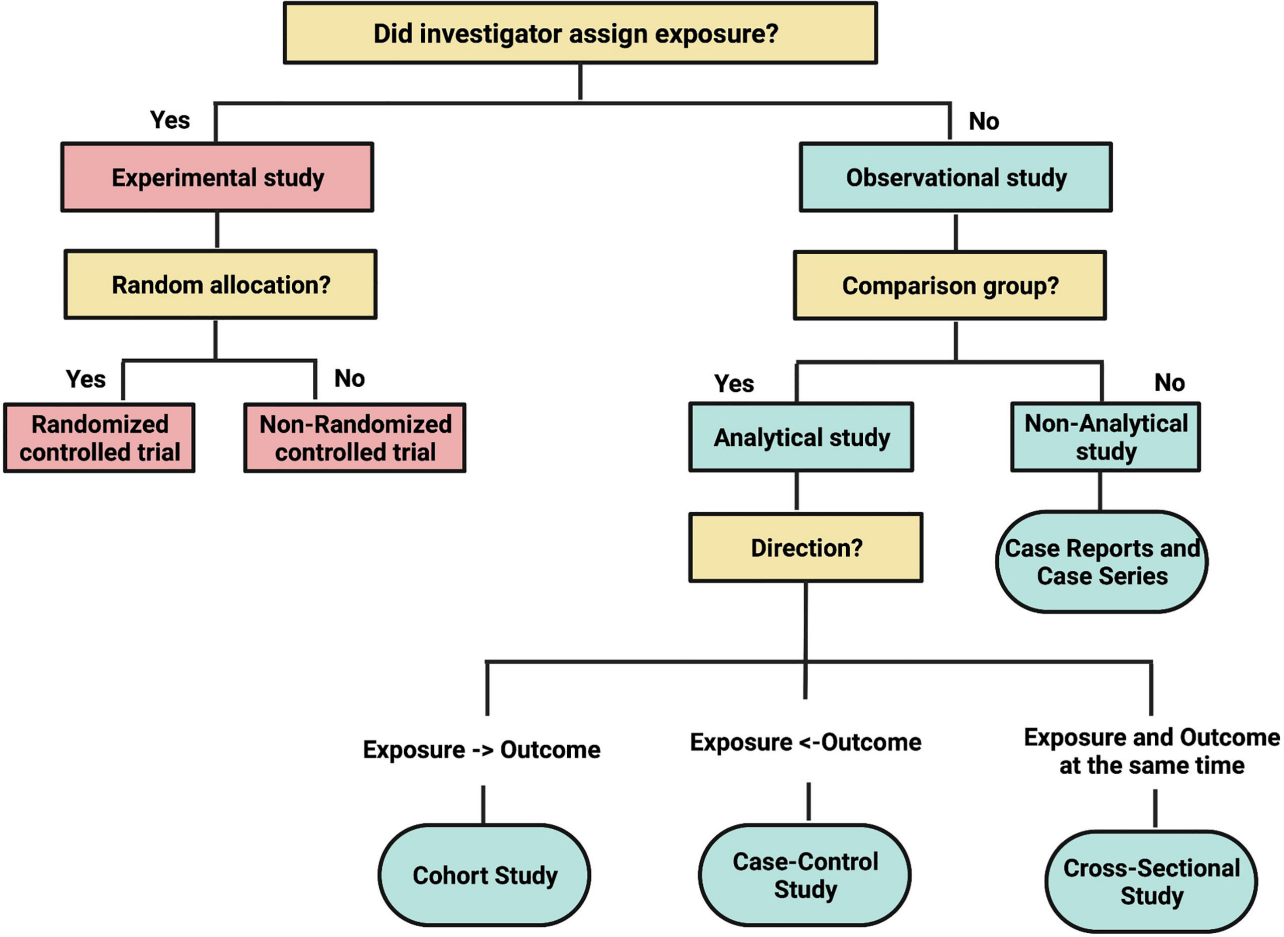
Adapted from *Lancet*. Grimes DA, and Schulz KF. An overview of clinical research: the lay of the land:57–61, 2002.

As in biology, anatomy dictates physiology. The anatomy of a study determines what it can and cannot do. Biology has animal and plant kingdoms. Similarly, clinical research has two large kingdoms: experimental and observational research.^1^ A useful classification system for the different types of clinical research study designs is depicted in **Figure 1**, reproduced from a classic article by Grimes and Schulz, published in the Lancet.^2^ If the study investigator assigns the exposure or intervention, the study is considered *experimental* in design. If the assignment of exposure or intervention is not under the control of the study investigator, i.e., not for the purpose of a study protocol per se, then the study is *observational* in design.

## Experimental Study Design

### Randomized Controlled Trials

**Figure 2:**
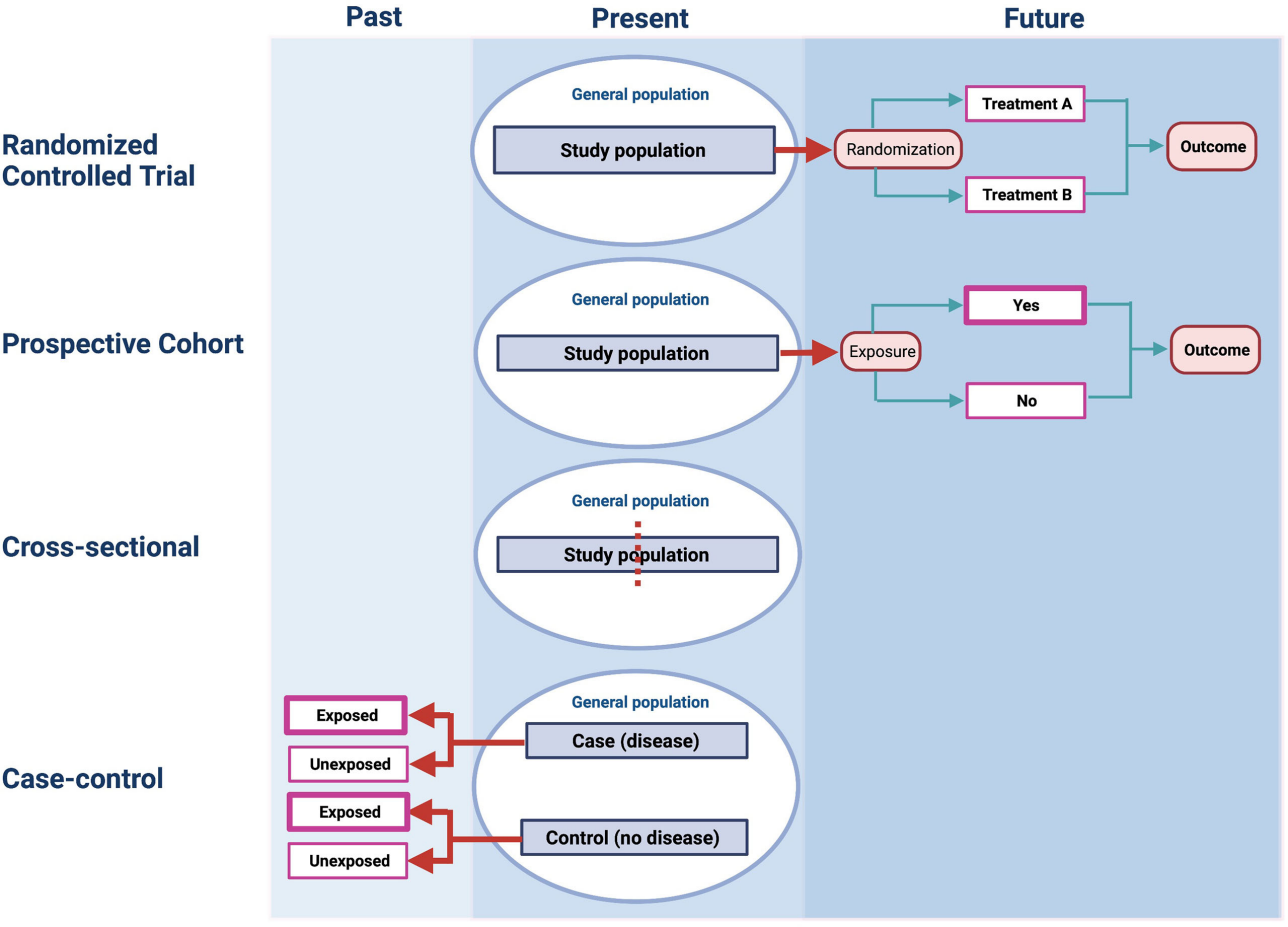
The structure of different study designs.

Once a study has been deemed experimental in design, the next step is to decide on the mechanism by which the intervention or exposure will be allocated (**Figure 2**). If it involves a process whereby every patient recruited into the study has a fixed probability of receiving the intervention or the comparator, then the study is called a *randomized controlled trial* (RCT). If a non-random mechanism (e.g., alternation) is used for allocation, then the study is called a *non-randomized controlled trial* (1). The latter design is uncommonly seen now since randomization is considered the gold standard mechanism for treatment allocation in a clinical trial setting.

The main outcome of randomization is that it reduces the likelihood that prognostic characteristics of the study patients will be unequally distributed between the intervention and comparator arms. As the sample size increases, the probability that important factors will be imbalanced across treatment groups will further decrease. Moreover, this uncoupling of the link between treatment allocation and patient prognosis ensures that, both *known* and *unknown* baseline characteristics of study patients will be balanced across treatment groups (3, 4, 13).

#### Strengths of Randomized Controlled Trials

RCTs are the ideal study type to investigate the benefit or harm of an intervention, such as a drug therapy (13, 14). When properly implemented, random allocation precludes selection bias, since inclusion and exclusion criteria are applied equally across all patients eligible for randomization and prior to treatment allocation (15, 16). A unique strength of this study design is that it eliminates confounding bias, both known and unknown, at least at the point of randomization. This design approximates the controlled experiment of basic science research. The hallmark of the RCT is assignment of participants to exposures purely by the play of chance. RCT are an excellent study design for producing results with high internal validity.

#### Limitations of Randomized Controlled Trials

Although RCTs are powerful tools, they also have some weaknesses. RCTs are not a panacea (17). In a number of situations, RCTs are impossible, inappropriate, inadequate, or unnecessary (18). Randomized trials are expected to be free only from baseline confounding. However, post-randomization confounding and selection bias can emerge in randomized trials (19). Moreover, patients may be differentially lost to follow up or drop out of the study before their outcome is ascertained and patients may not adhere to the assigned treatment (20).

Numerous important health exposures simply cannot be randomized, either for practical or ethical reasons. For instance, exposure to radiation and cigarette smoke cannot be randomized. Ethical objections may prevent interventions to be tested within an RCT setting when a well-accepted best practice is compared with treatment with an unknown or potentially less favorable outcome (3, 17). The results of RCTs may have low generalizability. RCTs tend to be conducted in selected patient populations due to their restrictive inclusion and exclusion criteria. Whereas the RCT, if properly done, has internal validity - i.e., accurately estimate causal effects within the group of participants in the study - it may have less external validity when applying the results to a larger population of real-world patients (21).

The problem of generalizability has practical implications the design and interpretation of RCTs. Unlike the observational study, the RCT often includes only volunteers who pass through a screening process before inclusion. Those who volunteer for trials tend to be different from those who do not; for example, their health might be better. In addition, it is well known that people may act differently when they are being observed (i.e., Hawthorne effect). The results from a closely monitored trial population may not accurately reflect what will happen when an intervention is moved into a general population. To mitigate this problem, knowledge of the level of exposure assigned to each group should be withheld from subjects and their providers (they are “blinded”), when possible (14).

Finally, RCTs are generally more expensive to conduct than observational studies (3, 4, 17). As a result, the duration of follow-up may insufficient to detect rare adverse events or measure the frequency of events that take many years to develop. To investigate these types of events, large-scale cohort studies or case–control studies are needed.

#### Example of a Randomized Controlled Trial in Kidney Transplantation

**Figure 3:**
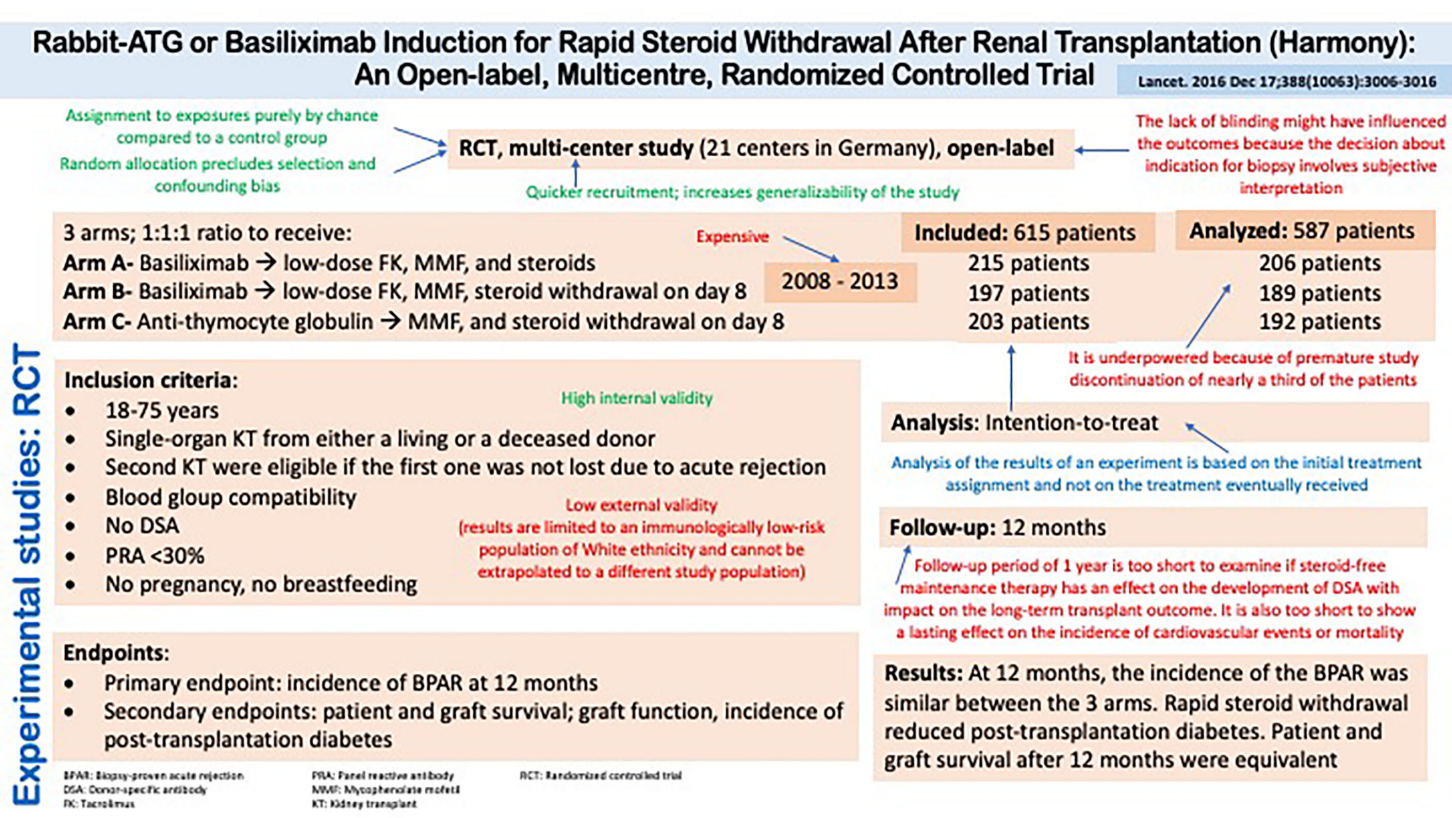
Experimental studies: Randomized Controlled Trial. Rabbit-ATG or Basiliximab Induction for Rapid Steroid Withdrawal After Renal Transplantation (Harmony): An Open-label, Multicentre, Randomized Controlled Trial.

**Figure 3** summarizes the design, conduct, and results of the Harmony study, which is a randomized controlled trial comparing rabbit anti-thymocyte globulin vs. basiliximab induction in patients undergoing rapid steroid withdrawal after kidney transplantation (22). It highlights various characteristics of the trial that may enhance or reduce the validity of the inferences that can be made from the results, as well as highlighting some notable features of the study.

## Observational Studies

Observational studies on the effects of therapy are usually the first step to generate and test hypotheses, but they may be more prone to biases than RCTs such that causal inferences must be made carefully. However, many observational studies do not focus on the effects of therapy but rather seek to answer research questions about etiology, diagnosis, prognosis, or adverse effects, areas where RCTs may be inappropriate or even impossible. The following the main types of observational study designs and a schema to classify them relative to each other is depicted in **Figure 1**.

### Descriptive Studies: Case Reports and Case Series

An observational study with no comparison group, for example patient(s) exposed to a novel treatment, is considered a *descriptive* study. This is the domain of case reports and case series where the outcomes of patients on a novel treatment may be described.

#### Strengths of Descriptive Studies

Case reports and case series can provide the basis for more rigorous, hypothesis-driven, analytical studies to examine the mechanism of disease, effect of a novel intervention, or the emergence of a new/rare adverse event from exposure to a specific drug or risk factor. Other potential roles of case reports and case series include the recognition and description of new diseases, medical education, and highlighting rare manifestations of common conditions (3, 23).

#### Limitations of Descriptive Studies

An important caveat of descriptive studies is that it does not allow for assessments of associations between treatment/exposure and disease since there is no comparison group. Only comparative studies (both experimental and analytical observational) enable assessments of possible causal relationships (1, 14).

#### Example of a Descriptive Study

**Figure 4:**
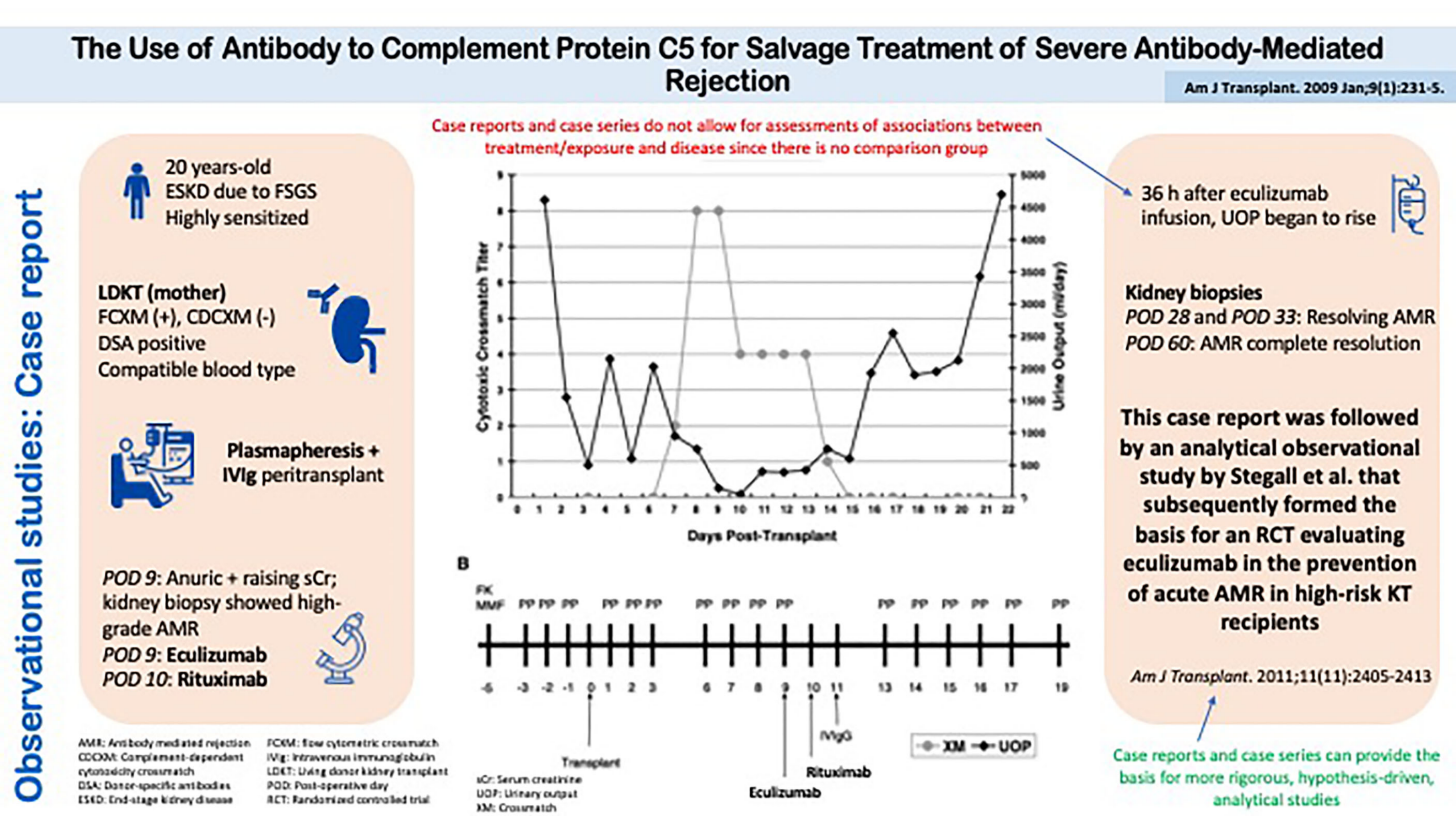
Observational studies: Case Report. The Use of Antibody to Complement Protein C5 for Salvage Treatment of Severe Antibody-Mediated Rejection.

A descriptive study that has led to the design of more definitive analytical studies is the case report by Locke et al. (**Figure 4**) (24). This report described a patient with refractory acute antibody-mediated rejection who, upon treatment with eculizumab, had an improvement in kidney function and histology, both of which returned to baseline within two months of treatment. This report was followed by an analytical observational study by Stegall et al. that has subsequently formed the basis for an RCT evaluating eculizumab in the prevention of acute antibody-mediated rejection in high-risk kidney transplant recipients (25).

### Analytical Observational Studies

There are three major types of analytical observational study designs in clinical research: cohort, case-control, and cross-sectional studies (**Figure 1**).

#### (a) Cohort Studies

In a cohort study, patients from a population are recruited into the study using clearly defined and prespecified inclusion/exclusion criteria. At the time of recruitment, an exposure or risk factor, such as delayed graft function or current smoking, is measured in each patient and the development of the outcome or disease in the “exposed” group is examined against a comparable group of patients who are “unexposed” (1, 14, 17).

Since all patients entering a cohort study are free of the outcome or disease of interest at the time of study recruitment, the *incidence* or new case rate of a disease over follow-up can be estimated from the cohort. The time from study entry, i.e., the time when patients come under observation, to the time of the outcome of interest can be measured in cohort studies. Therefore, this is the only study design (along with RCTs) that permits survival analysis. Importantly, only patients at risk for the outcome of interest should be included in a cohort study. For example, a study of risk factors for recurrent acute rejection should only include patients who have already had one episode of acute rejection (26).

Cohorts of patients may be assembled in the present and followed into the future for the event(s) of interest. This is known as a *prospective* cohort study (**Figure 2**). Alternatively, existing datasets may be used to assemble cohorts from the past and then track the occurrence of the outcome over time. This is known as a *retrospective* cohort study. Both designs are fundamentally cohort studies since they assemble patients at some clearly defined time point (either in the present or past) and then follow them forward in time to measure the outcome of interest (26). The structure of a prospective cohort study shown **Figure 2** is identical to a retrospective cohort study except the latter defines cohort entry by study participants at some point in the past and the outcomes are ascertained forward in time towards the present.

Retrospective cohort studies are often assumed to be inherently inferior to prospective cohort studies in terms of study validity. This is not necessarily true. The key issue is the quality and breadth of data collection and adherence to good study design principles. An existing dataset may not have been meant to answer a specific scientific question, but if the relevant data have been comprehensively captured, the rigor and quality of a retrospective cohort study can rival any prospective cohort study (27). A key issue in the design of retrospective cohort studies is to avoid the use of variables collected after cohort entry to inform the inclusion or exclusion of study patients. For example, the development of acute rejection should not be used as the basis for exclusion of patients in a cohort study examining the association of delayed graft function on the risk of death with graft function in kidney transplant recipients (28).

##### Strengths of Cohort Studies

Cohort studies are the best way to ascertain both the incidence and natural history of a disorder. Cohort studies are analytical studies that have the potential to provide answers to research questions on interventions, etiology (e.g., smoking, alcohol, or genetic factors), diagnosis, and prognosis. They are useful in the investigation of multiple outcomes that might arise after a single exposure. A prototype would be cigarette smoking (the exposure) and stroke, emphysema, oral cancer, and heart disease (the outcomes). The cohort design is also useful in the study of rare exposures, such as the health effects of ionizing radiation or chemicals in the workplace (29).

Cohort studies can reduce the risk of survivor bias if study entry criteria are equally applied across exposure groups (15). Diseases that are rapidly fatal are difficult to study because of this factor. For example, a hospital-based case control study of the linking snow-shovelling and myocardial infarction would miss all those who died in the driveway. A cohort study would be a less biased (but more cumbersome) approach: compare rates of myocardial infarction among those who shovel and those who do not shovel (26).

The strengths of cohort studies further lie in their potentially larger sample sizes, as a result of their lower cost compared to RCTs and in their broader patient populations resulting in a higher generalizability of their results. Recent work has highlighted design features in cohort studies that may allow for the emulation of a target trial (30). This approach may increase the confidence with which we may infer causality about treatment effects evaluated in cohort studies. Cohort studies on the effects of therapy may also generate hypotheses and provide an indication for the effect size, which is necessary for sample size calculations in RCTs; both will assist in the design of subsequent RCTs. In this respect, RCTs largely depend on work from preceding observational studies (2, 15, 17, 31).

##### Limitations of Cohort Studies

Cohort studies have important limitations too. Selection of certain patient population is built into cohort studies. For example, in a cohort study investigating effects of jogging on cardiovascular disease, those who choose to jog probably differ in other important ways (such as diet and smoking) from those who do not exercise (15). Whether these differences lead to *selection bias* depend on how study entry criteria are defined for exposed and unexposed individuals.

Cohort studies can be inefficient because it may take a long time before an outcome occurs and, as a result, these studies can be expensive (2). The cohort design is not optimal for rare diseases (e.g., posttransplant lymphoproliferative disease) or those that take a long time to develop (e.g., cancer). However, long-standing registries may accumulate a sufficient number of these less common events to allow for meaningful analyses. Loss to follow-up can be a challenge for study validity, even over the short-term but it can be particularly problematic in longitudinal studies that continue for decades (26).

##### Example of a Cohort Study

**Figure 5:**
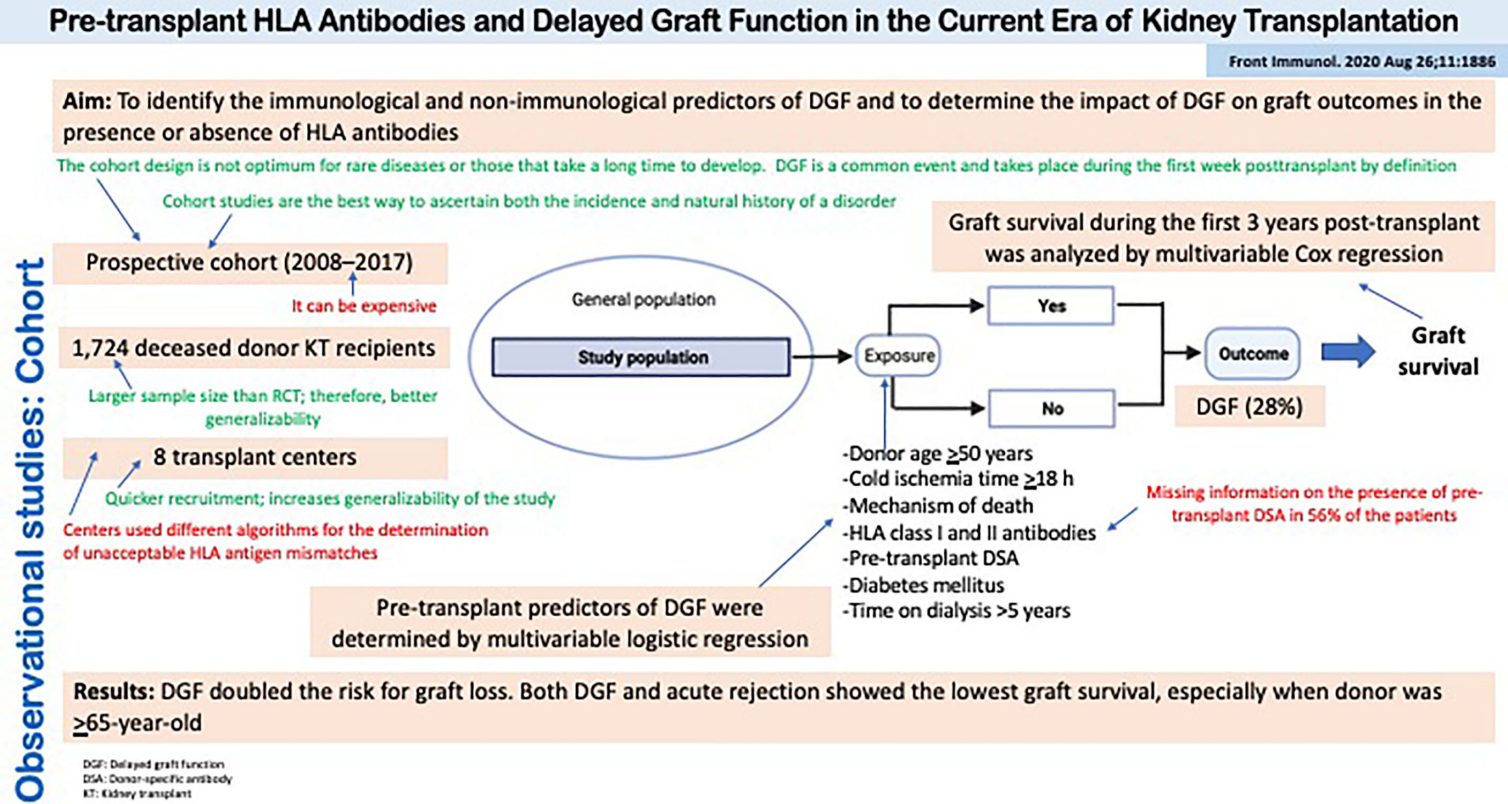
Observational studies: Cohort. Pre-transplant HLA Antibodies and Delayed Graft Function in the Current Era of Kidney Transplantation.

**Figure 5** summarizes the design, conduct, and results of a cohort study evaluating the role of HLA antibodies on the risk of delayed graft function and the impact of the latter on graft outcomes in the presence or absence of HLA antibodies (32). The presence of preformed HLA antibodies or delayed graft function cannot be randomized to kidney transplant recipients, so the observational cohort study design is best suited to address this question of etiology. Moreover, the outcome is the time to an event of interest (i.e., graft loss), thus survival analysis techniques applied to a cohort of patients must be used.

#### (b) Case-Control Studies

Case-control studies are less common in the transplant literature but are widely used in other areas such as genetic epidemiology. Case-control studies initially assemble patients based on their outcome (i.e., diseased) and then group of patients who did not have the outcome (i.e., non-diseased) are sampled from the population of interest. Subsequently, the exposure status of diseased patients at some time point in the past is ascertained and compared to the exposure status among non-diseased patients. Inherently, case-control studies are retrospective in the sense that both the exposure and outcome have already occurred by the time the study is conceived. Note that the case-control study is still an analytical observational study since the investigator did not assign the exposure or risk factor and there are at least two comparison groups (2, 31).

Five main notions guide investigators who do, or readers who assess, case-control studies. First, investigators must explicitly define the criteria for ascertainment of a case and any eligibility criteria used for selection. Second, controls should come from the same population as the cases, and their selection should be independent of the exposures of interest. Third, investigators should blind the data gatherers to the case or control status of participants or, if not possible, at least blind them to the main hypothesis of the study. Fourth, data gatherers need to be thoroughly trained to elicit or collect data on exposures in a similar manner from cases and controls. Finally, investigators should address the potential for confounding bias at both the design and analysis stages (31).

##### Strengths of Case-Control Studies

The case-control study is the most efficient design for evaluating rare diseases or outcomes that take many years to develop since it takes advantage of existing datasets with exposures and outcomes that have already been captured (1, 2, 31). Case control studies often require less time, effort, and money than cohort studies (2). They also may require smaller sample sizes than the equivalent cohort study (3, 17).

##### Limitations of Case-Control Studies

Although easier to do, they are also easier to do wrong. The Achilles heel of case-control studies is choosing an appropriate control group. Control patients should be individuals who are eligible to develop the disease in question but were disease-free at the time of control selection. This pool of patients is also known as the *study base* or *source population* (since it is the source of the cases) (1, 31). However, this population may be difficult to determine in advance and thus the approach to control selection requires careful thought and execution. Additionally, errors in measurement of exposure status must be considered and addressed. For example, recall bias (where there is better recollection of exposures among the cases than controls, or vice versa) may adversely impact the validity of the study results (14, 15).

##### Example of a Case-Control Study

**Figure 6:**
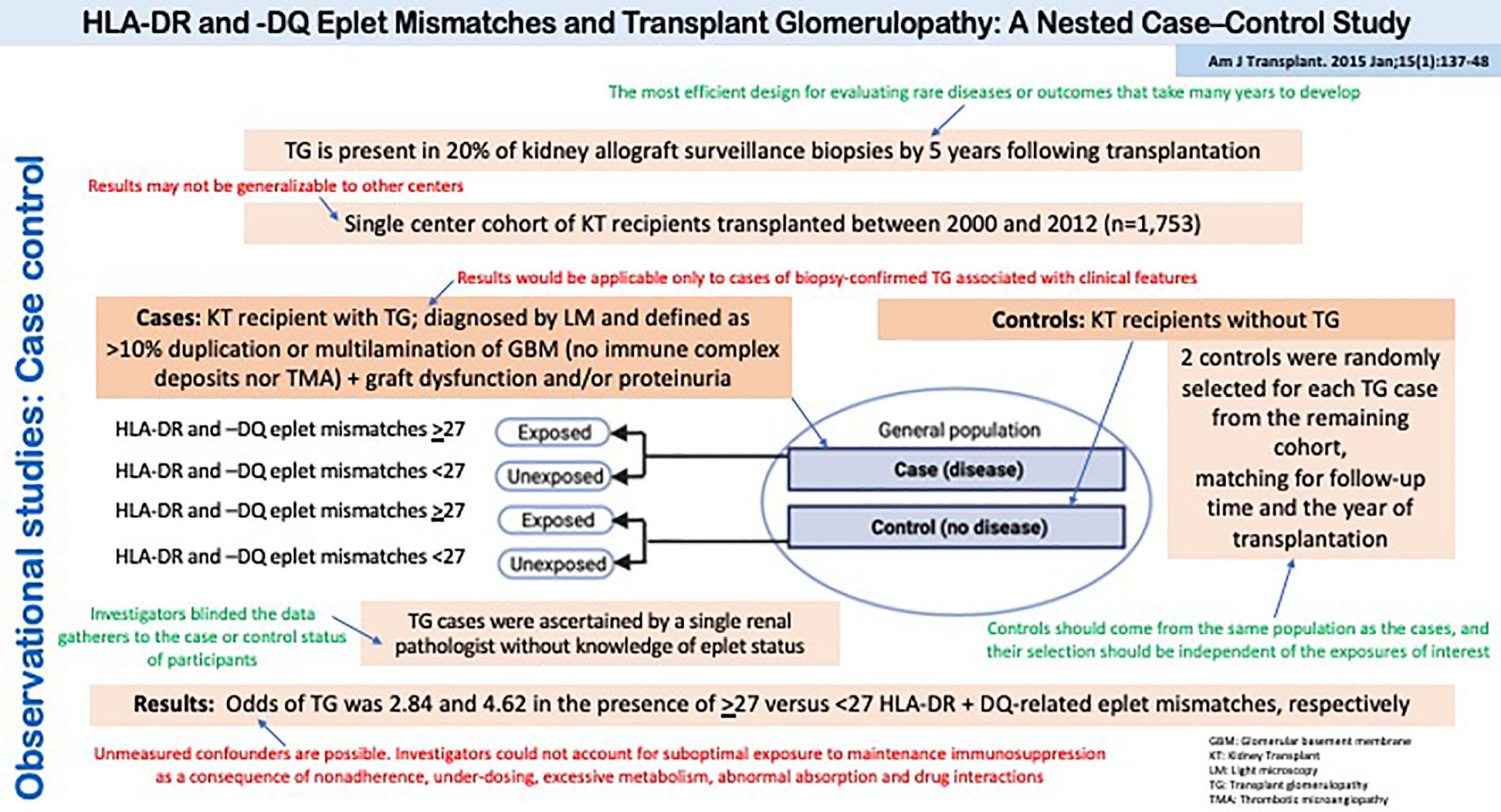
Observational studies: Case Control. HLA-DR and -DQ Eplet Mismatches and Transplant Glomerulopathy: A Nested Case–Control Study.

**Figure 6** depicts a study by Sapir-Pichhadze et al. that used a case-control design to evaluate the role of DR and DQ eplet mismatches on the development of transplant glomerulopathy (33). Of note, the study was nested within a well-defined cohort of kidney transplant recipients in a single center in Toronto, Canada, which allowed the investigators to clearly define the study base or source population from which the cases were derived (and thus, the source for control selection). This is also known as a nested case-control study.

#### (c) Cross-Sectional Studies

In cross-sectional studies, the exposure and outcome is usually assessed at the same time. This hampers the interpretation of associations because the temporality of exposure and outcome may be uncertain. Therefore, this study design can draw limited causal inferences about relationships between exposure and outcome. Having said this, since patients can be categorized into exposure groups, and the proportion of patients with the outcome of interest can be calculated among exposed and unexposed patients, this design qualifies as an analytical observational study.

Cross-sectional studies can be thought of as providing a ‘snapshot’ of the frequency and characteristics of an outcome at a particular point in time. In general, cross-sectional studies are prevalence studies (number of cases existing per 1000 population) but cannot describe incidence (number of new cases per unit time). As a result, their findings can be used to estimate the burden of diseases in populations for the planning of health services delivery (5, 17).

##### Strengths of Cross-Sectional Studies

Cross-sectional studies can be performed quickly, since no follow-up is necessary, and at little expense. It can also provide clues to scientifically interesting associations that may be later confirmed in cohort studies or an RCT. As a result, cross-sectional analyses have been typically referred to as *hypothesis-generating* studies. As previously noted, the absence of follow-up precludes estimation of the incidence rate. Instead, the *prevalence* (i.e., the proportion of patients with the outcome or disease at a given time) is the main metric of interest, while the prevalence of disease in the exposed versus unexposed is the main comparison of interest (1, 2, 17).

##### Limitations of Cross-Sectional Studies

The major weakness of cross-sectional studies is that the temporal relation between exposure and outcome may not be clearly delineated and thus associations derived from these studies may be susceptible to *reverse causality*. The latter refers to situations where the outcome has affected the exposure such that the association measured in a cross-sectional study may be biased. For example, a survey of patients’ current smoking habits and a history of lung cancer may erroneously suggest that smoking is protective against lung cancer. However, the development of lung cancer may have altered the taste for cigarettes leading to a reduction in smoking frequency versus the non-cancer group (1).

##### Example of a Cross-Sectional Study

**Figure 7:**
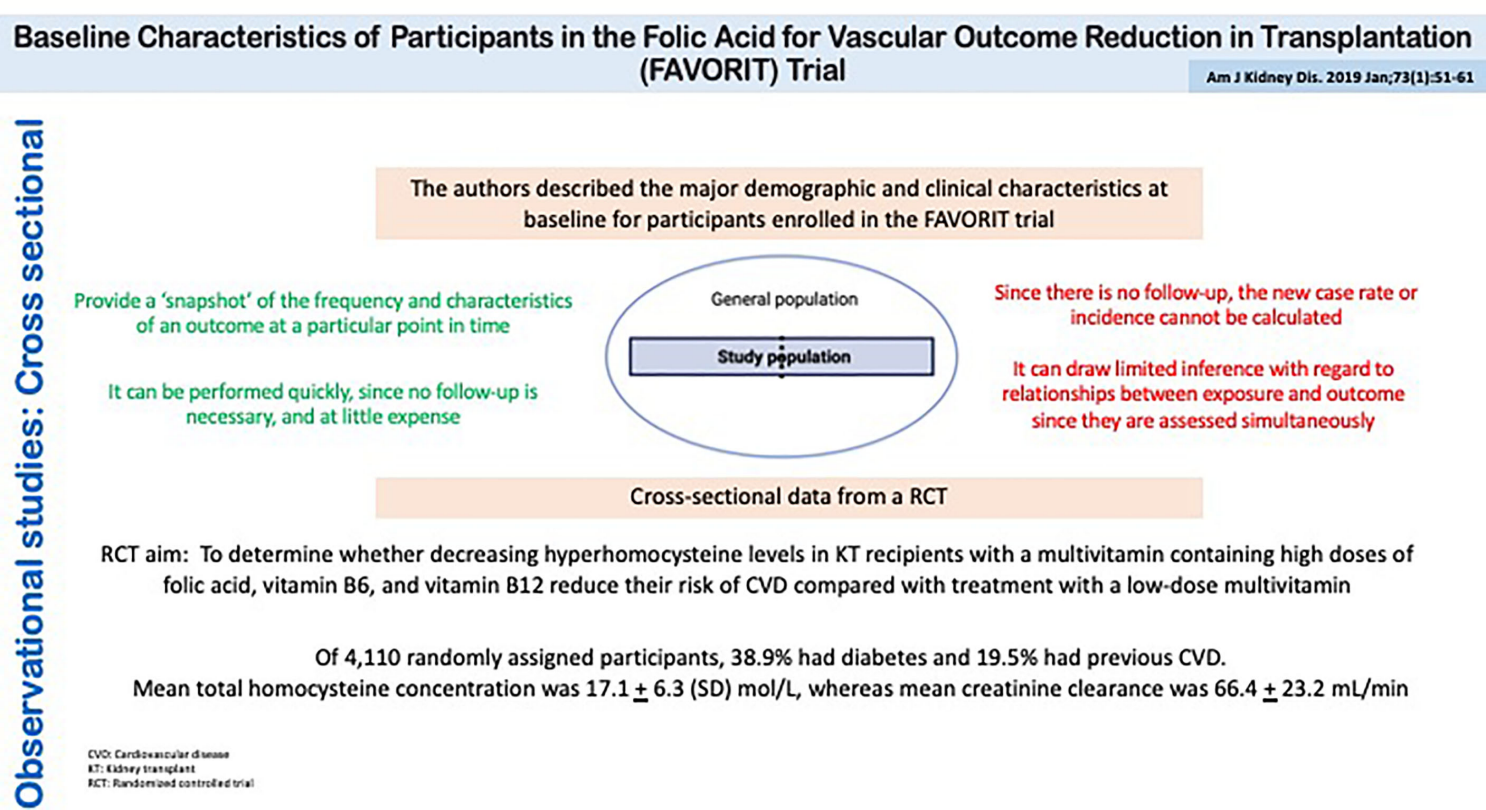
Observational studies: Cross Sectional. Baseline Characteristics of Participants in the Folic Acid for Vascular Outcome Reduction in Transplantation (FAVORIT) Trial.

Cross-sectional analyses of baseline characteristics are commonly performed in clinical trial populations. **Figure 7** shows a cross-sectional study performed on the patients recruited for the FAVORIT trial which assessed the role of folic acid in potentially reducing vascular outcomes after kidney transplantation (34). These types of analyses serve to characterize the trial population, with the goal of assessing its comparability to kidney transplant recipients in clinical practice. Moreover, one could evaluate baseline correlates of elevated homocysteine levels. The association of baseline factors with treatment should be null given the latter’s random allocation.

## Other Study Designs – Meta-Analysis

Meta-analysis is a statistical method that quantitatively combines results from different studies to come up with a pooled estimate of the treatment or exposure effect. It is typically conducted on clinical trials that address the same intervention but can also be used to pool other types of data, such as studies on diagnostic accuracy (e.g., pooled estimates on sensitivity and specificity) and epidemiologic studies (e.g., pooled incidence or prevalence rates) (35, 36). The main advantage of a meta-analysis is the ability to derive a more precise estimate of treatment/exposure effects by effectively increasing the sample size. However, a well-done meta-analysis of poorly designed studies will yield invalid results. Bias and confounding in the primary studies are significant problems for meta-analysis. Moreover, a meta-analysis itself can be poorly executed (36, 37). For example, the inappropriate selection of studies (due to inappropriate inclusion/exclusion criteria or publication bias) may lead to biased effect estimates.

## Linking Clinical Questions to Study Designs

Classifying studies by their design characteristics is helpful in highlighting the methodologic features that each one possesses to support causal inferences, while outlining features that may influence their susceptibility to bias. However, sometimes the problems that clinicians face make the linkage between a given question and the appropriate study design less than obvious. Herein, several clinical scenarios are described that lead to specific research questions. The types of study designs that most appropriately address the questions are also provided. **Table 1** outlines the types of questions and their associated study designs.

### Questions About Interventions

Ms. Gonzalez has end-stage secondary to lupus nephritis. She received a kidney transplant 28 days ago and has developed antibody-mediated rejection treated with plasmapheresis, intravenous immunoglobulin (IVIg) and corticosteroids (i.e., standard of care). Her father has read that eculizumab is a drug that potentially may help her daughter. However, the use of this medication might be associated with severe infections and is rather expensive.

The patient’s physician asks the following question: In kidney transplant recipients, does the addition of eculizumab (vs. no eculizumab), in patients treated with plasmapheresis, IVIg, and corticosteroids for antibody-mediated rejection, improve kidney transplant outcomes? Given that this is classic question of treatment efficacy, the optimal study design would be an RCT. However, a multi-center cohort study may also be pursued if eculizumab has been variably used across different transplant centers for the indication of antibody-mediated rejection, along with a background of standard of care for all patients. Theoretically, a case-control study could also be conducted, although they are used less commonly for questions of treatment efficacy.

### Questions About Etiology

Cardiovascular mortality is the leading cause of death in patients with end-stage kidney disease. In addition to traditional risk factors, proinflammatory cytokines, C-reactive protein, and T cell-mediated immunity have been shown to relate to cardiovascular morbidity and mortality in dialysis patients. A transplant nephrologist has two patients on hemodialysis waiting for a kidney transplant, with the same age, race, and dialysis vintage. Neither patient has diabetes, prior transplants, nor autoimmune diseases, but the first patient has cPRA of 0%, while the second patient has cPRA of 98%.

The patient’s physician asks the following question: Among end-stage kidney disease patients on hemodialysis awaiting a kidney transplant, does a higher level of cPRA (vs. a lower level) independently predict the future risk of cardiovascular mortality? This is a question of disease etiology. Since cPRA cannot be randomized, an RCT would not be appropriate. Cardiovascular mortality occurs over follow-up. As a result, the cohort design is most suitable for answering this question. A case-control study may be preferred over a cohort study if additional data collection was necessary (e.g., abstracting cPRA data from HLA reports), since data collection would only have to performed for cases and a sample of controls.

### Questions About Diagnosis

Fibrillary glomerulonephritis (FGN) is a rare primary glomerular disease. Histologic and histochemical features of FGN overlap with those of other glomerular diseases and no unique histologic biomarkers for diagnosing FGN have been identified. A novel FGN-specific diagnostic marker called DNAJB9 has been touted as a potentially useful clinical tool to diagnose FGN. Mr. Jones originally kidney disease is unknown but has now developed a glomerular disease in his kidney transplant. The histologic features are suggestive of FGN but there remains some diagnostic uncertainty.

The patient’s physician asks the following question: In kidney transplant recipients with suspected FGN, what are the operating characteristics (i.e., sensitivity, specificity, predictive value) of DNAJB9 in diagnosing FGN? Diagnostic studies are cross-sectional since the biomarker being evaluated is applied to a group of patients, some with and some without disease (as per the gold standard test of disease).

### Questions About Prognosis

Mr. Smith developed end-stage kidney disease secondary to autosomal dominant polycystic kidney disease and is undergoing a living donor kidney transplant next week. He has an impaired glucose tolerance test and has been informed that he may develop post-transplant diabetes. He would like to know the probability of developing posttransplant diabetes over the first post-transplant year, given his age, oral glucose tolerance test result, and other characteristics.

The patient’s physician asks the following question: In patients with a history of autosomal dominant polycystic kidney disease who undergo a kidney transplant, what is the likelihood of being diagnosed with post-transplant diabetes in the first year after kidney transplantation, as a function of their glucose tolerance and other baseline factors? Studies of prognosis are typically evaluating outcomes that occur at some follow-up time after patients come under risk for the outcome in question. As a result, all prognosis studies require cohort designs.

### Questions About Describing Disease Burden

Frailty, a measure of physiologic reserve, is associated with poor outcomes and mortality among kidney transplant candidates and recipients. The director of a kidney transplant program would like to offer all her patients the best and most comprehensive care possible. When she requests for the allocation of resources to rehabilitate frail patients, she is asked how often this problem occurs in her transplant center.

The physician asks the following question: Among kidney transplant candidates and recipients followed at our center, how frequently are patients diagnosed with frailty? Depending on its intended uses, the best measure of disease frequency can either be prevalence or incidence. The former may be estimated cross-sectionally in a group of kidney transplant candidates and recipients at one point in time. However, if the director would like to understand the future needs of patients being followed in her program, a measure of incidence (i.e., new cases of frailty) would be most appropriate. The latter can only be estimated from a cohort study.

## Other Important Considerations

The quality of a clinical study depends on internal and external factors. Studies have internal validity when, random error apart, reported differences between exposed and unexposed individuals can be attributed only to the exposure under investigation. Internal validity can be affected by two types of error: random error and systematic error. Random error depends on chance and can be minimized by increasing the sample size. Systematic errors are flaws in study design and/or analysis that can lead to an over- or under-estimation of the association of interest. This type of error is independent of sample size. Systematic error is also known as bias (15, 38).

Selection, information, and confounding biases are the three major forms of bias in clinical research. Although these biases are typically discussed in the context of observational studies, similar problems can arise in experimental studies. Selection bias stems from an absence of comparability between groups being studied. Information bias results from errors in the measurement of exposure, outcome, and/or confounders. Confounding is a mixing or confusion of effects; a researcher attempts to relate an exposure to an outcome but actually measures (at least in part) the effect of a third factor, i.e., the confounding variable. Bias can be prevented at two levels: (1) by choosing the appropriate study design for addressing the study hypothesis and (2) by carefully establishing the procedures of data handling and the definitions of exposures and outcomes. **Table 2** outlines the main considerations for bias in observational studies. Several accessible review of bias in clinical research exist in the medical literature (38).

**Table 2:** How to look for bias in observational studies.

**SELECTION BIAS** ***Cohort Studies*** Were the same criteria used for study entry among groups defined by exposure? Were all patients accounted for over follow-up? Was one exposure group more likely to have losses to follow-up that were driven by factors impacting the likelihood of developing the outcome?
***Case-Control Studies*** Were controls selected independent of their exposure status? Were controls sampled from the underlying cohort from which cases were derived?
**INFORMATION BIAS** ***Cohort Studies:*** Is information about outcome obtained in the same way for those exposed and unexposed? ***Case-Control Studies:*** Is information about exposure gathered in the same way for cases and controls?
**CONFOUNDING BIAS** ***Cohort and Case-Control Studies*** Could the results be accounted for by the presence of a factor –e.g., age, smoking, sexual behaviour, diet – associated with both the exposure and the outcome but not directly involved in the causal pathway?

In recent years, new observational study design methods, that take advantage of the evolving causal inference literature (39, 40), have been developed to improve the validity of treatment comparisons using observational data. This method emulates a target trial using observational data and has been shown to derive estimates of treatment effects that are comparable to clinical trials and in contrast to studies using more conventional observational study designs (27, 30). These observational studies can help to extend the findings of clinical trials and/or provide estimates of treatment effects in populations that were not studied in the original clinical trials (41, 42). They can also effectively leverage existing large data sources such as electronic health records and population-based administrative datasets.

## Conclusions

Clinical research can be divided into experimental and observational; observational studies are further categorized into those with and without a comparison group. Only studies with comparison groups allow investigators to assess possible causal associations. The RCT remains the gold standard study design to evaluate the effects of therapies. In contrast, studies of etiology, diagnosis, prognosis, or disease burden rely heavily on observational designs. Usually, results from observational studies are needed to generate hypothesis that can subsequently be tested within an RCT. They can also be used to corroborate the results of RCTs in real-world settings and ascertain long-term outcomes that could not be observed over the usual follow-up duration of an RCT. Both observational studies and RCTs fulfill a complementary and valuable role in transplant.

## Author Contributions

SR-R wrote the first draft of manuscript. SR-R and SK performed the literature review. SR-R designed the figures and tables. SK revised the manuscript. All authors contributed to the article and approved the submitted version.

## Conflict of Interest

The authors declare that the research was conducted in the absence of any commercial or financial relationships that could be construed as a potential conflict of interest.

The handling Editor [WL] declared a past collaboration with the author [SK].

## Publisher’s Note

All claims expressed in this article are solely those of the authors and do not necessarily represent those of their affiliated organizations, or those of the publisher, the editors and the reviewers. Any product that may be evaluated in this article, or claim that may be made by its manufacturer, is not guaranteed or endorsed by the publisher.
